# Combining conventional ultrasound and ultrasound elastography to predict HER2 status in patients with breast cancer

**DOI:** 10.3389/fphys.2023.1188502

**Published:** 2023-07-12

**Authors:** Xiaoying Zhuo, Ji Lv, Binjie Chen, Jia Liu, Yujie Luo, Jie Liu, Xiaowei Xie, Jiao Lu, Ningjun Zhao

**Affiliations:** ^1^ Ultrasound Medicine Department of the Affiliated Hospital of Xuzhou Medical University, Xuzhou, China; ^2^ Medical Imaging College of Xuzhou Medical University, Xuzhou, China; ^3^ Emergency Medicine Department of the Affiliated Hospital of Xuzhou Medical University, Xuzhou, China; ^4^ College of Computer Science and Technology, Jilin University, Changchun, China; ^5^ Pathology Department of the Affiliated Hospital of Xuzhou Medical University, Xuzhou, China; ^6^ Laboratory of Emergency Medicine, Second Clinical Medical College of Xuzhou Medical University, Xuzhou, China

**Keywords:** HER2, breast cancer, machine learning, shap, ultrasound

## Abstract

**Introduction:** Identifying the HER2 status of breast cancer patients is important for treatment options. Previous studies have shown that ultrasound features are closely related to the subtype of breast cancer.

**Methods:** In this study, we used features of conventional ultrasound and ultrasound elastography to predict HER2 status.

**Results and Discussion:** The performance of model (AUROC) with features of conventional ultrasound and ultrasound elastography is higher than that of the model with features of conventional ultrasound (0.82 vs. 0.53). The SHAP method was used to explore the interpretability of the models. Compared with HER2– tumors, HER2+ tumors usually have greater elastic modulus parameters and microcalcifications. Therefore, we concluded that the features of conventional ultrasound combined with ultrasound elastography could improve the accuracy for predicting HER2 status.

## 1 Introduction

Breast cancer is one of the most common malignancies in women (Harbeck and Gnant, 2017). It is estimated that there were 2.26 million new cases of breast cancer worldwide in 2020 (Sung et al., 2021). Breast cancer is a highly heterogeneous tumor. Common molecular subtypes of breast cancer include luminal A (LA), luminal B (LB), human epidermal growth factor receptor 2 over-expression (HER2+) and triple negative breast cancer (TNBC), and different molecular subtypes show significant differences in biological behavior, clinical outcome and patient prognosis (Lüönd et al., 2021). Among these molecular subtypes, HER2+ patients make up about 15%–20% of all breast cancer cases and shows high malignancy, high rate of recurrence and metastasis, and poor prognosis (Guarneri et al., 2013). In recent years, trastuzumab (an antibody that targets HER2) (Hudis, 2007) has been used in clinical practice, and the prognosis of HER2+ patients has improved significantly (Kümler et al., 2014). It shows that accurate identification of the molecular subtype of breast cancer is essential for treatment. The 2018 American Society of Clinical Oncology/American Association of Pathologists Detection Guide and 2019 Chinese breast cancer HER-2 Detection Guide regulate the IHC staining requirements and the interpretation of IHC and ISH result (Wolff et al., 2018). In this consensus, HER-2 IHC 3+or HER-2 IHC 2+/ISH+ is defined as HER-2 positive, IHC 1+or IHC 2+/ISH–is defined as HER-2 low expression, and IHC 0 is defined as HER-2 negative.

So far, identification of HER2+ mainly relies on fluorescence *in situ* hybridization (FISH) and immunohistochemistry (IHC) (Baez-Navarro et al., 2023). However, the two methods are invasive procedures and may lead to seroma (Ebner et al., 2018) and infection (Bruening et al., 2010). Therefore, we need non-invasive, economical and accurate methods to predict HER2 status in breast cancer.

Ultrasound imaging technologies are non-invasive, convenient and affordable and have been widely used for breast cancer screening and diagnosis (Berg et al., 2015). It has been shown that ultrasonographic features are related to molecular subtypes of breast cancer (Wu et al., 2019; Gumowska et al., 2021). Many machine learning models for predicting molecular subtypes of breast cancer have been developed (Zhou et al., 2021; Ma et al., 2022). However, these models mainly relied on the characteristics of conventional ultrasound. In recent years, the development of ultrasound elastography (Barr, 2018) has provided new opportunities for breast cancer screening and diagnosis (Carlsen et al., 2015; Yao et al., 2023). As a new imaging technology, ultrasonic elastic imaging can evaluate the hardness of the lesions and thus identify the nature of the lesions, which is an important supplement to traditional ultrasonic imaging. At present, the ultrasonic elastography technology used for breast diagnosis mainly includes strain elastography and acoustic palpation elastography. Sound touch elastography (STE) is a kind of ultrasonic imaging technology developed recently in China, which can display the tissue hardness information in the region of interest (ROI) in real time, and provide the elastic value related to the mass and its periphery through Shell quantitative analysis tool kit. The hardness change of the lesion tissue was measured accurately. However, to the best of our knowledge, there are no studies exploring the relations between characteristics of ultrasound elastography and HER2+. In this study, we build a machine learning model for HER2 status prediction based on the characteristics of conventional ultrasound combined with ultrasound elastography. In addition, Shapley additive explanations (SHAP) method (Lundberg et al., 2020; Lv et al., 2023) was used to explore the interpretability of the model. We hope that the model can provide more valuable information for personalized healthcare of breast cancer.

## 2 Materials and methods

### 2.1 Cohorts

Patients with breast cancer at the Affiliated Hospital of Xuzhou Medical University between January 2021 and December 2022 were used in this study. All patients were confirmed by gross needle aspiration biopsy or surgical pathology.

Exclusion criteria were as follows: 1) pregnant or lactating women; 2) tumor diameter more than 50 mm; 3) patients who have undergone interventional treatment (e.g., chemotherapy, radiotherapy) before ultrasound examination; 4) patients with severe organ insufficiency; 5) poor patient compliance. Finally, 51 patients with HER2+ breast cancer were enrolled in this study. As controls, we also recruited 52 patients with HER2-breast cancer and 50 patients with benign breast disease. The study follows the “Transparent Reporting of a Multivariable Prediction Model for Individual Prognosis or Diagnosis” (Collins et al., 2015). All patients were de-identified.

### 2.2 Ultrasound

Ultrasound scans were obtained using Mindray Resona 7S Doppler Color Ultrasound and a liner transducer L14-5WU with strain elastography and acoustic palpation elastography system. The operations and assessments were performed by three physicians skilled in ultrasound elastography and conventional ultrasound.

**FIGURE 1:**
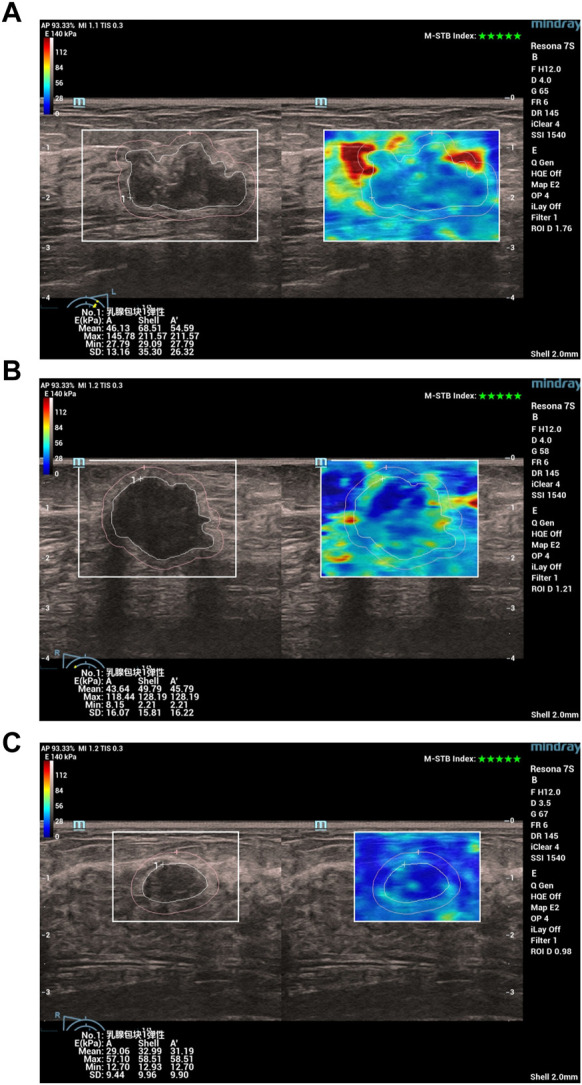
Examples of ultrasound elastography for **(A)** HER2+ breast cancer, **(B)** HER-breast cancer and **(C)** benign breast disease. Chinese characters 乳腺包块1弹性: in (A–C) stand for “Breast mass 1 elasticity“.

Specifically, all patients first underwent a conventional ultrasound examination. The location, size (maximum diameter), morphology, margins, orientation, echo pattern, microcalcification, and hyperechoic halo of the lesion were recorded. Next, the section with the most abundant blood flow was used to assess the blood flow classification (Adler classification (Adler et al., 1990)) and measured the resistance index (RI). Finally, all patients underwent an ultrasound elastography examination, strain ratio, strain elasticity score, lesion mean elastic modulus (A_mean_), lesion maximum elastic modulus (A_max_), lesion peripheral (shell 2 mm) mean elastic modulus (S_mean_), lesion peripheral maximum elastic modulus (S_max_) were recorded. In Figure 1, we show examples of ultrasound elastography for (a) HER2+ breast cancer, (b) HER-breast cancer and (c) benign breast disease.

### 2.3 Statistical analysis

Python (Version 3.7) was used for statistical analysis and visualization. One demographic feature, nine conventional ultrasound features, and six ultrasound elastography features were used in this study (Table 1; Table 2). Among these features, age, size, resistance index, strain elasticity score, strain ratio, A_mean_, A_max_, S_mean_, and S_max_ are continuous variables, while orientation, shape, margin, echo pattern, microcalcification hyperechoic halo and Adler classification are discrete variables. For continuous variables, they are presented as median ± interquartile range (IQR), and Mann-Whitney test was used for group comparisons (e.g., HER2+ breast cancer vs. HER2-breast cancer). For discrete variables, they are presented as count (percentage), and chi-square test was used for group comparisons. 2-sided *p*-value <0.05 was considered significantly different.

**TABLE 1:** The characteristic stratified by tumor status.

Characteristic	Breast cancer (*n* = 103)	Benign tumor (*n* = 50)	*p*-value
Demographics			
Age (year, mean ± SD)	51 ± 10.5	37 ± 11.75	1.02 × 10^−16^
Conventional ultrasound			
Size (cm, mean ± SD)	1.80 ± 1.00	1.40 ± 0.78	0.041
Orientation			0.015
Not parallel	14 (13.59%)	0	
Parallel	89 (86.41%)	50 (100%)	
Shape			1.1 × 10^−15^
Irregular	98 (95.15%)	17 (34%)	
Oval	5 (4.85%)	33 (66%)	
Margin			1.1 × 10^−20^
Not circumscribed	93 (90.29%)	6 (12%)	
Circumscribed	10 (9.71%)	44 (88%)	
Echo pattern			5.7 × 10^−12^
Heterogeneous	82 (79.61%)	10 (20%)	
Hypoechoic	21 (20.39%)	40 (80%)	
Microcalcification			3.3 × 10^−9^
Yes	54 (52.43%)	1 (2%)	
No	49 (47.57%)	49 (98%)	
Hyperechoic halo			0.011
Yes	22 (21.36%)	2 (4%)	
No	81 (78.64%)	48 (96%)	
Adler classification			7.4 × 10^−11^
1	3 (2.91%)	21 (42%)	
2	54 (52.43%)	25 (50%)	
3	46 (44.66%)	4 (8%)	
Resistance index	0.78 ± 0.04	0.62 ± 0.08	8.08 × 10^−23^
Ultrasound elastography			
Strain elasticity score	4 ± 0	3 ± 1	3.24 × 10^−18^
Strain ratio (%)	4.15 ± 0.76	2.61 ± 1.07	1.34 × 10^−21^
A_mean_ (kPa)	39.56 ± 10.74	28.52 ± 7.88	1.65 × 10^−16^
A_max_ (kPa)	90.48 ± 15.25	54.97 ± 14.06	1.92 × 10^−22^
S_mean_ (kPa)	51.24 ± 15.07	32.78 ± 8.55	3.95 × 10^−18^
S_max_ (kPa)	153.27 ± 49.49	65.69 ± 17.93	1.43 × 10^−23^

**TABLE 2:** The characteristic stratified by HER2 status.

Characteristic	HER2+ (*n* = 51)	HER2− (*n* = 52)	*p*-value
Demographics			
Age	53 ± 10	51 ± 11.25	0.436
Conventional ultrasound			
The size of mass (cm)	2 ± 1.05	1.6 ± 0.9	0.039
Orientation			0.804
Not parallel	7 (13.73%)	7 (13.54%)	
Parallel	44 (86.27%)	45 (86.54%)	
Shape			0.07
Irregular	51 (100%)	47 (90.38%)	
Oval	0	5 (9.62%)	
Margin			0.003
Not circumscribed	51 (100%)	42 (80.77%)	
Circumscribed	0	10 (19.23%)	
Echo pattern			0.056
Heterogeneous	45 (88.24%)	37 (71.15%)	
Hypoechoic	6 (11.76%)	15 (28.85%)	
Microcalcification			4.8 × 10^–7^
Yes	40 (78.43%)	14 (26.92%)	
No	11 (21.57%)	38 (73.08%)	
Hyperechoic halo			0.85
Yes	11 (21.57%)	11 (21.15%)	
No	40 (78.43%)	41 (78.85%)	
Adler classification			0.002
1	0	3 (5.77%)	
2	20 (39.22%)	34 (65.38%)	
3	31 (60.78%)	15 (28.85%)	
Resistance index	0.79 ± 0.04	0.76 ± 0.04	2.84 × 10^–5^
Ultrasound elastography			
Strain elasticity score	4.0 ± 1.0	4.0 ± 0.0	2.3 × 10^–6^
Strain ratio (%)	4.39 ± 0.96	4.06 ± 0.59	0.0055
A_mean_ (kPa)	40.33 ± 10.82	38.34 ± 9.40	0.026
A_max_ (kPa)	94.68 ± 41.43	89.66 ± 11.00	0.008
S_mean_ (kPa)	58.49 ± 15.81	48.49 ± 12.76	3.15 × 10^–6^
S_max_ (kPa)	170.99 ± 53.13	135.08 ± 43.40	0.0001
Pathology			0.184
IDC I	3 (5.88%)	9 (17.31%)	
IDC II	24 (46.06%)	23 (44.23%)	
IDC III	24 (46.06%)	20 (38.46%)	

IDC: invasive ductal carcinoma.

### 2.4 Machine learning models

A tree-based machine learning approach was used for feature selection (Ke et al., 2017). In the tree-based model, zero-importance features are not used to split any nodes, so the features have no impact on the performance of tree-based models. Previous study has shown that we can obtain the best results if 70%–80% of the data is used for training, and 20%–30% of the data is used for testing (Gholamy et al., 2018). Therefore, all patients were randomly divided into a training set (80%) and a test set (20%). The extreme gradient boosting (XGBoost) model (Chen and Guestrin, 2016) was used to predict the status of tumor (benign tumor or breast cancer) and the status of HER2 (HER2+ or HER2−). Hyperparameters of models (e.g., n_estimators, max depth, learning rate) were selected by *k*-fold cross-validation on the training set. Usually, *k* is set to 5 or 10. However, the size of dataset used in this study is small, and a larger *k* leads to larger fluctuations in the performance of the model (Supplementary Figure S1). Therefore, *k* is set to 5. The model with the optimal hyperparameters was validated by the holdout test set, and area under the receiver operating characteristic curve (AUROC) was used to evaluate the performance of models. The 95% confidence interval of AUORC on test set was calculated by 1000 bootstrap replicates. The SHAP method was used to explore interpretability of models (Lundberg et al., 2020).

In addition, we also developed a logistic regression (LR) model to predict the status of HER2. We then compared performance of the LR model with that of the XGBoost model.

## 3 Results

### 3.1 Cohort characteristics

The cohort included 51 patients with HER2+ breast cancer, 52 patients with HER2-breast cancer and 50 patients with benign breast disease. For patients with breast cancer and benign breast disease, all characteristics showed significant differences (Table 1). Therefore, all features were used to predict the status of tumor (breast cancer or benign tumor). However, for patients with HER2+ breast cancer and HER2-breast cancer, age, orientation, shape, echo pattern, hyperechoic halo and pathology did not show significant differences (Table 2). In addition, we used a tree-based machine learning model (i.e., LightGBM) to calculate the importance of the features. As shown in Supplementary Table S1, orientation, shape, margin, echo pattern, hyperechoic halo and Adler classification are zero importance features. In tree-based machine learning models, the features do not have any effect on the performance of models. Therefore, microcalcification, A_mean_, resistance index, S_mean_, A_max_, S_max_, size and strain ratio were used to predict the status of HER2 (HER2+ or HER2−). Subsequently, we explored whether the features of conventional ultrasound combined with ultrasound elastography could improve the predicted accuracy of tumor status and HER2 status.

### 3.2 Prediction of tumor status

**FIGURE 2:**
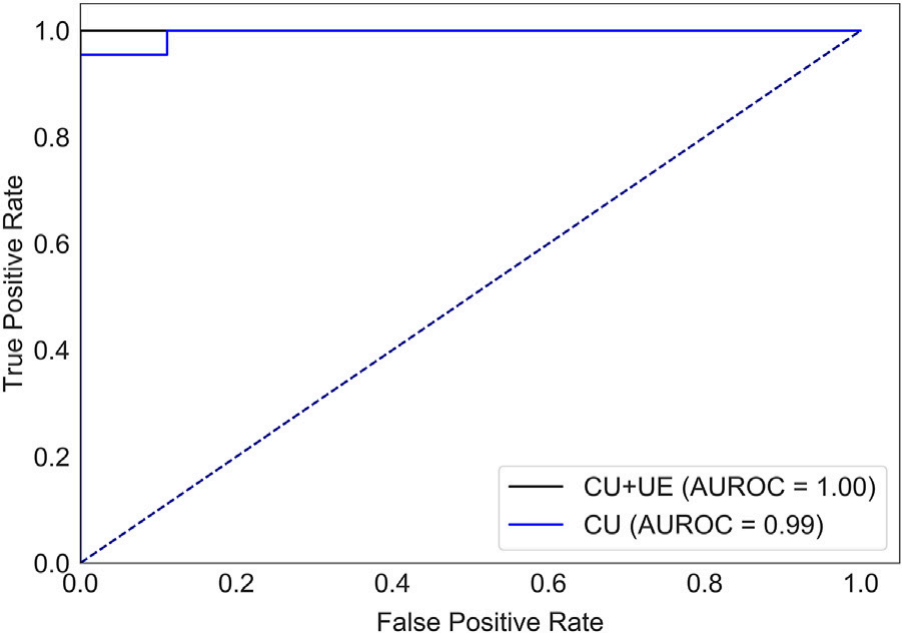
AUROCs of tumor status prediction models with ultrasound elastography features and/or conventional ultrasound features for the test set. CU: conventional ultrasound. UE: ultrasound elastography.

There were 82 patients with breast cancer and 40 patients with benign breast disease in the training set, and there were 21 patients with breast cancer and 10 patients with benign breast disease in the test set. All features (Table 1) were used to predict the status of tumor (breast cancer or benign tumor). For the model with features of conventional ultrasound, the cross-validation AUROCs ranged from 0.98 to 1 ( 0.99±0.01 , Supplementary Figure S2A), and the corresponding AUROC of the test set (95% CI) was 0.99 (0.97–1). For the model with features of conventional ultrasound and ultrasound elastography, the cross-validation AUROC ranged from 0.97 to 1 ( 0.99±0.01 , Supplementary Figure S2B), and the corresponding AUROC of the test set (95% CI) was 1.00 (1.00–1.00). AUROCs of the models with features of ultrasound elastography and/or conventional ultrasound are close to 1. One possible reason for this is that the test set held is a “too good” subset. To rule out this reason, the training set and test set were repeatedly split 10 times, and we report more evaluation metrics (i.e., sensitivity, specificity, negative predictive value and positive predictive value). The averaged AUROC, sensitivity, specificity, negative predictive value and positive predictive value of the model with features of conventional ultrasound are 0.996±0.009 , 0.967±0.036 , 0.935±0.059 , 0.972±0.024 , 0.934±0.074 , respectively. The averaged AUROC, sensitivity, specificity, negative predictive value and positive predictive value of the model with features of conventional ultrasound and ultrasound elastography are 0.997±0.006 , 0.975±0.025 , 0.960±0.089 , 0.988±0.025 , 0.956±0.045 , respectively. Overall, both models can predict the status of tumor accurately (Figure 2).

### 3.3 Prediction of HER2 status

**FIGURE 3:**
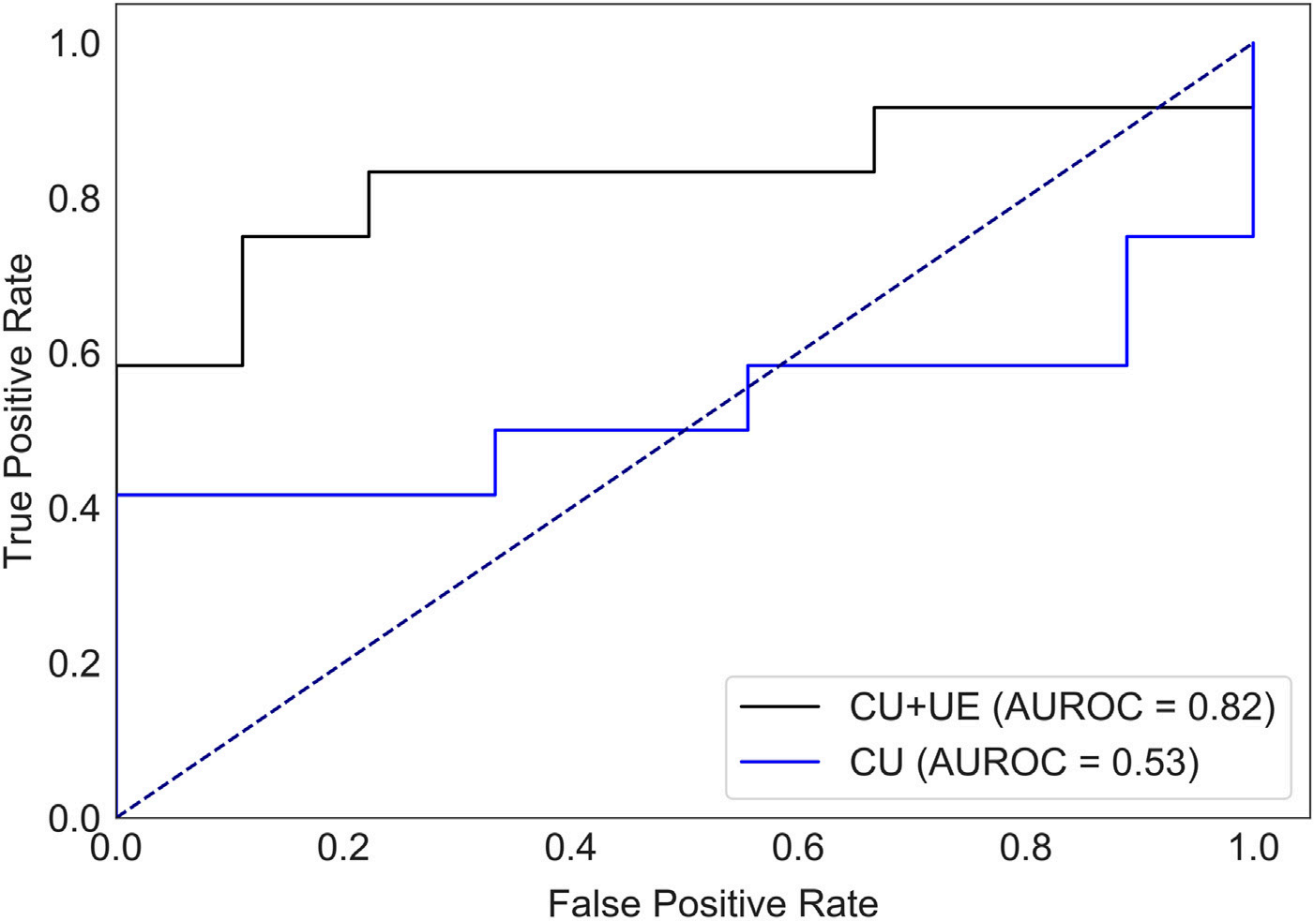
AUROC of HER2 status prediction models with ultrasound elastography features and/or conventional ultrasound features for the test set. CU: conventional ultrasound. UE: ultrasound elastography.

There were 40 patients with breast cancer and 41 patients with benign breast disease in the training set, and there were 11 patients with breast cancer and 11 patients with benign breast disease in the test set. As shown in Table 2, age, orientation, shape, echo pattern, hyperechoic halo and pathology did not show significant differences. Therefore, these features were not used to build machine learning models. For the model with features of conventional ultrasound, the cross-validation AUROC ranged from 0.53 to 0.93 ( 0.74±0.13 , Supplementary Figure S3A) and the corresponding AUROC of the test set (95% CI) was 0.53 (0.27–0.78). For the model with features of conventional ultrasound and ultrasound elastography, the cross-validation AUROC ranged from 0.69 to 0.88 ( 0.81±0.07 , Supplementary Figure S3B), and the corresponding AUROC of the test set (95% CI) was 0.82 (0.62–0.99). Therefore, we concluded that the features of conventional ultrasound combined with ultrasound elastography could improve the prediction accuracy of HER2 status (Figure 3).

Supplementary Table S1 provides valuable insights into the stepwise variable selection method. Next, we compared the performance of models with different features (i.e., top 8 features, top 10 features and top 16 features). As shown in Supplementary Figure S4, the model with the top 8 features showed the best performance. Introducing irrelevant features into the model can degrade the performance of the model.

To evaluate the performance of the XGBoost model, we also developed a LR model using the same training set and test set. Compared with the XGBoost model (Supplementary Table S2), LR model had a lower test AUROC (XGBoost mode, 0.82 vs. LR model, 0.72), lower precision (XGBoost mode, 0.88 vs. LR model, 0.80), higher recall (XGBoost mode, 0.58 vs. LR model, 0.67) and higher F1-value (XGBoost mode, 0.70 vs. LR model, 0.73). For HER2+ prediction, we prefer to screen out more suspected HER2+ patients than to miss a possible HER2+ patient, so the F1-value should be preferred as an evaluation metric. Therefore, LR model is a better choice for our prediction purposes (higher recall). However, for clinical prediction models, while the performance of the model is very important, the interpretability of the model should not be neglected. In recent years, the XGBoost model combined with the SHAP method have been widely used in cohort studies (Deng et al., 2022; Lv et al., 2023). These interpretable machine learning models can give not only the prediction results, but also the reasonable reasons for the judgments. Therefore, we prefer to use the XGBoost model. Next, we use SHAP model to explore the interpretability of the model.

### 3.4 Interpretability of the model

**FIGURE 4:**
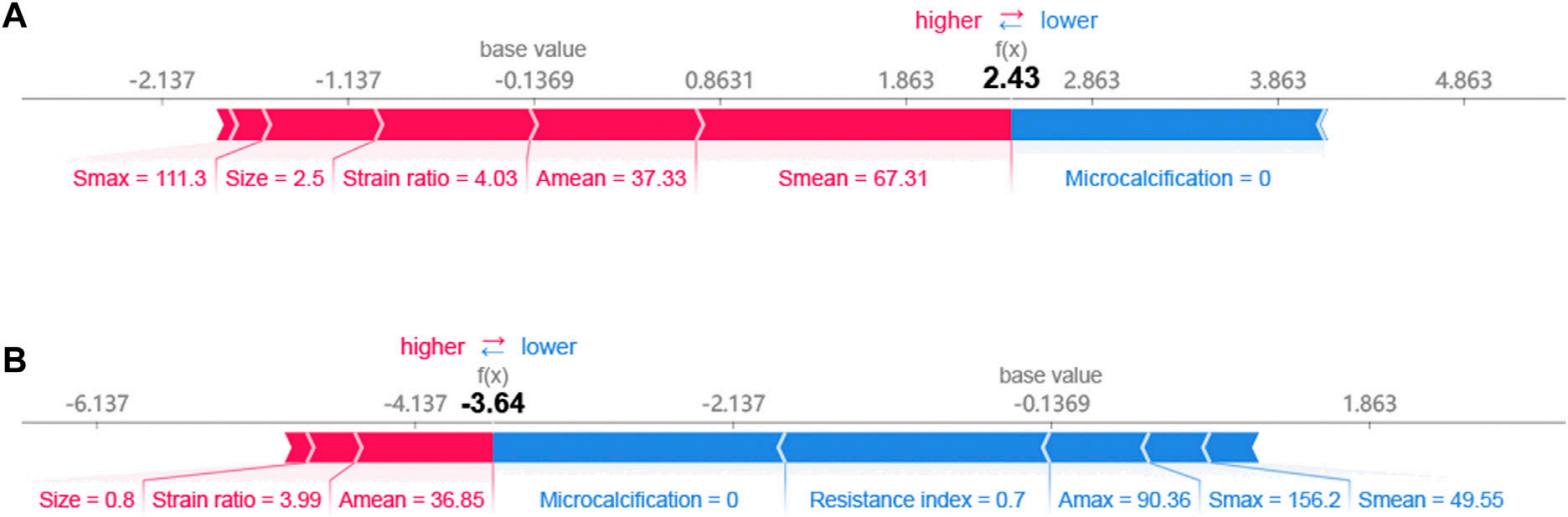
Breast cancer patients with **(A)** the highest and **(B)** the lowest SHAP values.

The SHAP method can help us identify key factors for HER2+ at the patient level and at the cohort level. First, we identified key factors for HER2+ at the patient level. As shown in Figure 4, We show a patient with the highest SHAP value (Figure 4A) and a patient with the lowest SHAP value (Figure 4B). The baseline is the mean SHAP value of −0.1369. The predicted risk for the patient with the highest SHAP value is 2.43. Microcalcification, larger S_mean_ (67.31) and so on are potential key factors for HER2+. For the patient with the lowest SHAP value (−3.64), no microcalcifications, lower resistance index and A_max_ and so on contribute to HER2−.

**FIGURE 5:**
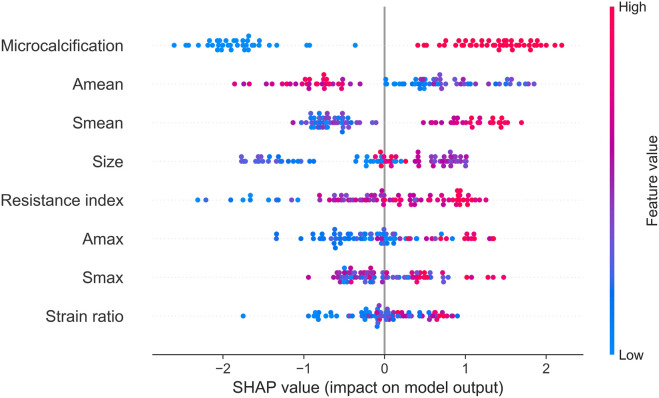
SHAP summary plot of the 8 key factors.

Next, we identified key factors for HER2+ at the cohort level. As shown in Figure 5, microcalcification, A_mean_, S_mean_, size and resistance index are the top 5 key factors to identify HER2 status. Compared with S_max_ and A_max_, A_mean_, and S_mean_ are better key factors to identify HER2 status.

**FIGURE 6:**
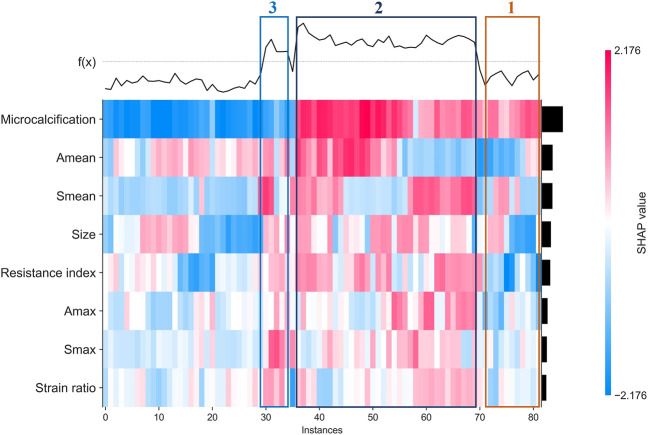
Heatmap that identify clusters of breast cancer patients who have similar characteristics and outcomes.

Finally, we used clustering algorithm to explore relations between these features. As shown in Figure 6, patients with similar features and similar subtypes were grouped together. Overall, microcalcifications have a strong correlation with HER2+ (cluster 2). However, smaller tumor and A_mean_ have a negative effect on the result of model (cluster 1). For patients without microcalcification, larger S_mean_ or S_max_ (cluster 3) increase the likelihood of HER2+. In addition, we also performed partial regression analysis. As shown in Supplementary Figures S5–S12, the effects of microcalcification, resistance index and S_mean_ on HER2+ were more significant. It shows that conventional ultrasound combined with ultrasound elastography can predict HER2 status better.

## 4 Discussion

Compared with other subtypes of breast cancer, HER2+ breast cancer is more malignant, more aggressive, and more likely to recur and metastasize (Guarneri et al., 2013). In recent years, the development of HER2-targeted drugs have led to significant benefits for patients with HER2+ breast cancer (Kümler et al., 2014). Therefore, it is critical to identify the HER2 status of breast cancer patients accurately and quickly.

Ultrasound is widely used for breast cancer screening and diagnosis (Berg et al., 2015), and previous studies have shown that there are some correlations between ultrasound characteristics and breast cancer subtypes (Wu et al., 2019; Gumowska et al., 2021). Conventional ultrasound can evaluate the shape, size, margin, and echo pattern of tumors. In summary, the shape of breast cancer lesions is irregular, the margin of the lesions is not circumscribed, the interior of the lesion is rich in blood flow, and the echo pattern is not homogeneous (Table 1). Both the machine learning model with conventional ultrasound and the machine learning model with conventional ultrasound and ultrasound elastography have shown excellent performance in predicting tumor status (Figure 2). However, machine learning models with conventional ultrasound haven shown moderate performance in predicting HER2 state (Figure 3). Ultrasound elastography can evaluate the hardness of tumors, providing a new opportunity for the prediction of HER2 status (Carlsen et al., 2015; Yao et al., 2023). The introduction of tumor elasticity information significantly improves the performance of the machine learning model (Figure 3). The SHAP method can help us identify key factors for predicting HER2 status (Figures 4–6).

For conventional ultrasound, size, margin, microcalcification, Adler classification and resistance index were considered as key factors for predicting HER2 status (Table 2; Figure 5). HER2+ stimulates the wild growth of cancer cells, leading to inadequate local blood supply, resulting in cell death and microcalcification (Zhou and Hung, 2003; Loibl and Gianni, 2017). Therefore, HER2+ tumors are usually larger and have microcalcifications (Table 2). In addition, HER2+ increases cancer cell aggressiveness (Pupa et al., 2021). Therefore, the margin of HER2+ are usually not circumscribed (Table 2). However, the prerequisite for rapid tumor growth and infiltration is the formation of a large number of microvessels (Furuya et al., 2005). Microvessels provide the nutrients and oxygen needed for tumor growth (Pluda and Parkinson, 1996). In this study, we found that HER2+ patients have a higher Adler classification (Table 2). This finding is consistent with previous studies (Pluda and Parkinson, 1996; Furuya et al., 2005).

For ultrasound elastography, we found that elastic modulus parameters (i.e., A_mean_, A_max_, S_mean_, and S_max_) were significantly higher in HER2+ tumors than in HER2-tumors (Table 2). It may be related to higher microvascular density and interstitial water in HER2+ tumors (Zhang et al., 2022; Kurt et al., 2023). Yoo et al. found that the hardness of the tumor is associated with tissue hypoxia (Yoo et al., 2020), and HER2 contributes to increased hypoxic response in breast cancer by regulating HIF-2α (Jarman et al., 2019). Therefore, we speculated that elastic modulus parameters of tumors can reflect the status of HER2 to some extent. In Figure 5, we found that microcalcification is the most important factor for predicting HER2 status, and it is consistent with the study of Elias et al. (Elias et al., 2014). However, there are some HER2+ patients without microcalcification. For the patients, elastic modulus parameters (i.e., S_mean_ and S_max_) can help us identify the HER2 status (Figure 6) and thus improve the performance of machine learning models (Figure 3).

Although this study is meaningful, our study still has some limitations: 1) This study is a retrospective single-center study with a small number of cases, and bias was inevitable; 2) The features used in this study were human-defined. With the development of deep learning, it is expected to automatically extract features from images (Lin et al., 2017; Banan et al., 2020).

## 5 Conclusion

In conclusion, ultrasound features are closely related to HER2 status. We developed interpretable machine learning models combined with conventional ultrasound and ultrasound elastography features to predict the state of HER2. The model combined with ultrasound elastography features showed better performance. Conventional ultrasound combined with ultrasound elastography can predict HER2 status better. Microcalcification, A_mean_, S_mean_, size and resistance index are the top 5 key factors to identify HER2 status. It is meaningful for breast cancer screening and diagnosis and personalized medicine.

## Data Availability

The raw data supporting the conclusion of this article will be made available by the authors, without undue reservation.
