# Supplementary material for: Combining conventional ultrasound and ultrasound elastography to predict HER2 status in patients with breast cancer
Source: Front Physiol. 2023 Jul 12;14:1188502. doi: 10.3389/fphys.2023.1188502 (PMC10369848; doi:10.3389/fphys.2023.1188502)
Supplement: Supplementary file 1 [file DataSheet1.docx]

Supplementary Material

Combining conventional ultrasound and ultrasound elastography to predict HER2 status in patients with breast cancer

# Supplementary Figures and Tables

## Supplementary Figures


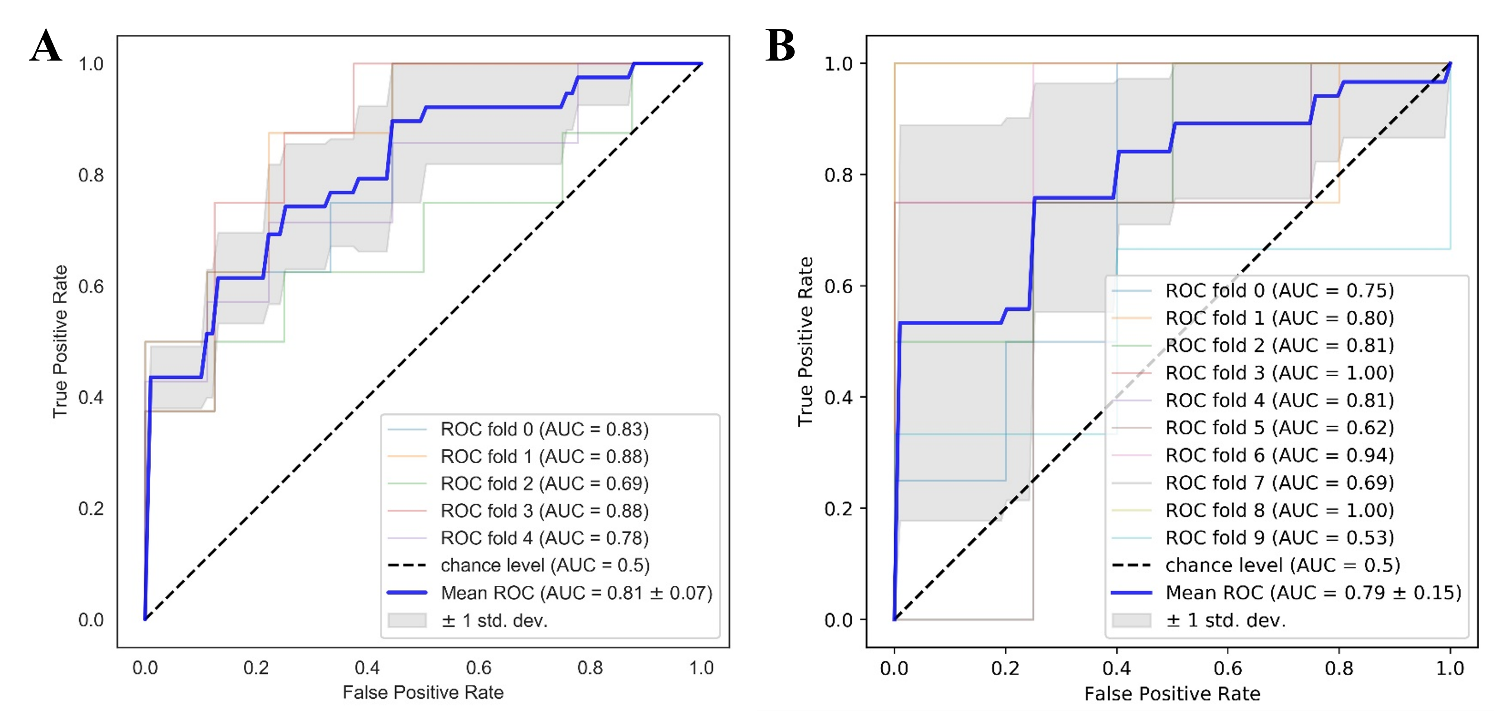


**Supplementary Figure S1.** AUROC of HER2 status prediction models with conventional ultrasound and ultrasound elastography features for the *k*-fold cross-validation set (A) *k* = 5 (B) *k* =10.


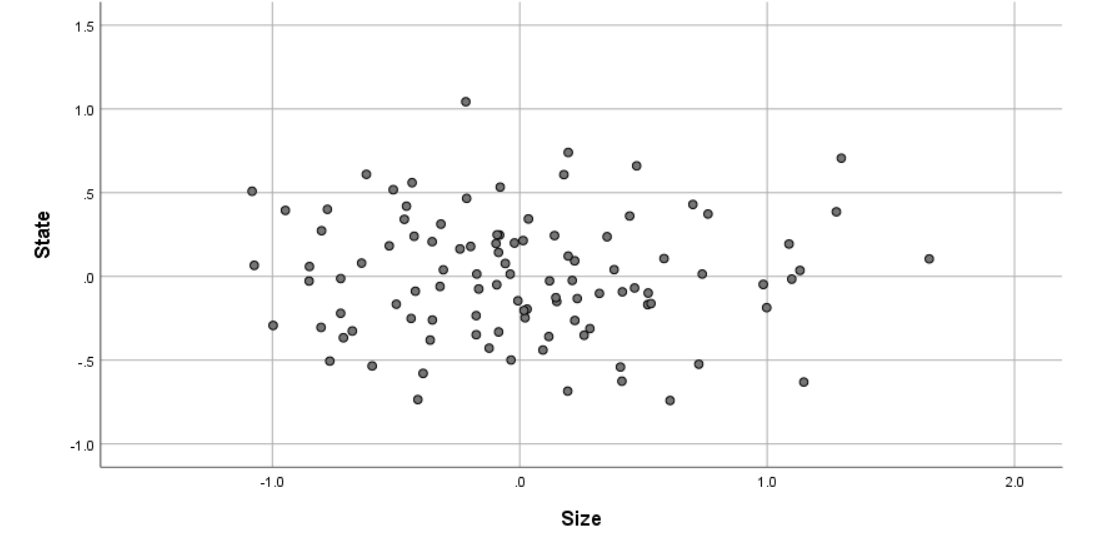
**Supplementary Figure S2.** Partial regression plot of size.


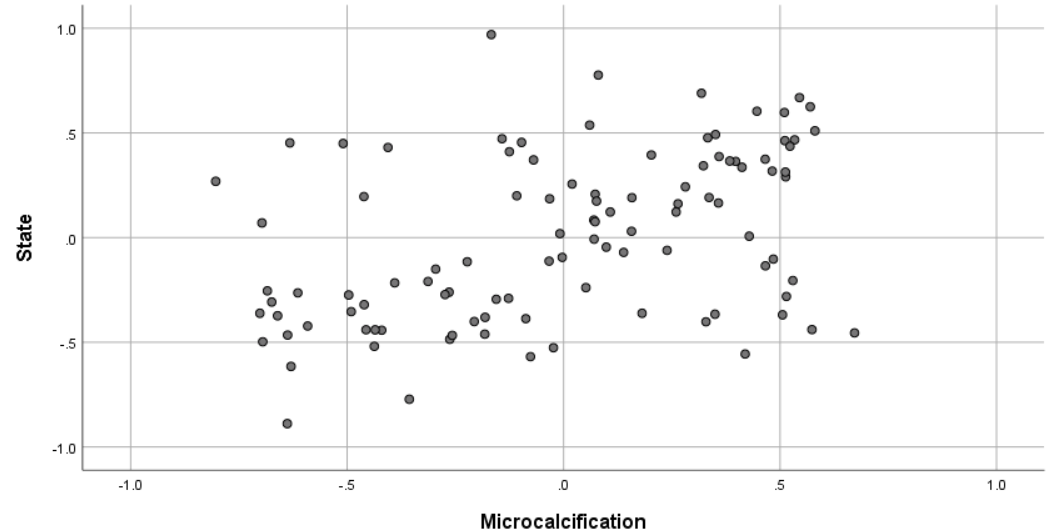


**Supplementary Figure S3.** Partial regression plot of microcalcification.


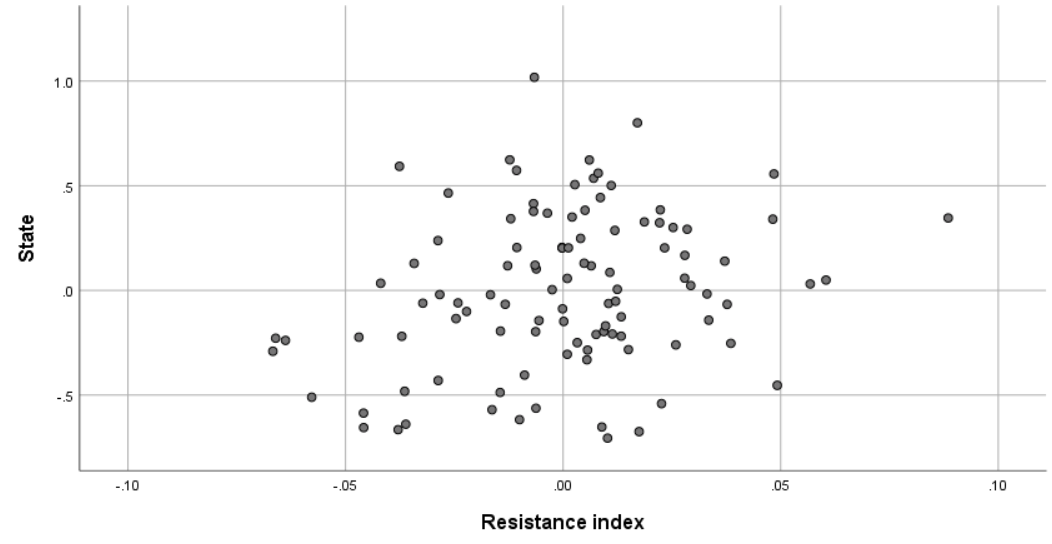


**Supplementary Figure S4.** Partial regression plot of resistance index.


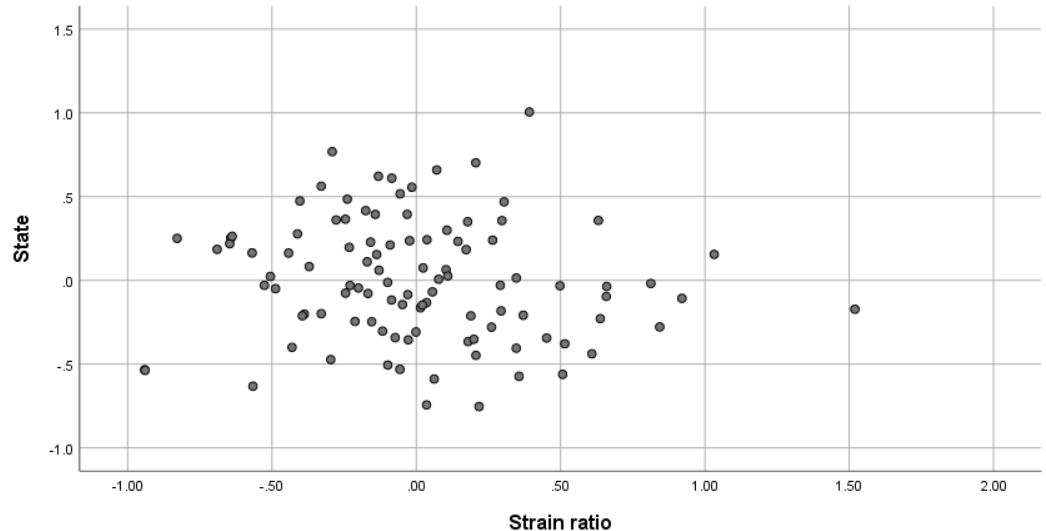


**Supplementary Figure S5.** Partial regression plot of strain ratio.


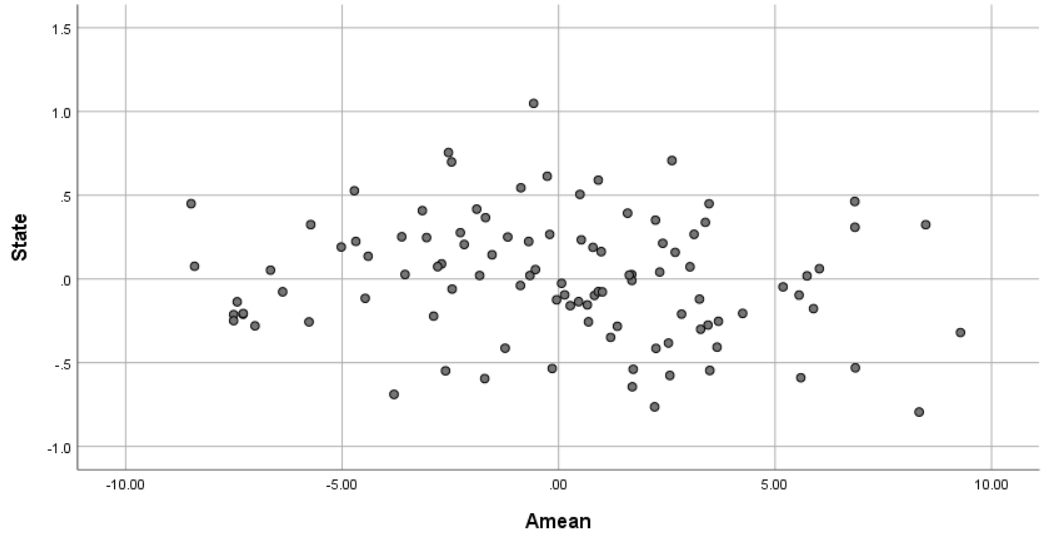


**Supplementary Figure S6.** Partial regression plot of A_mean_.


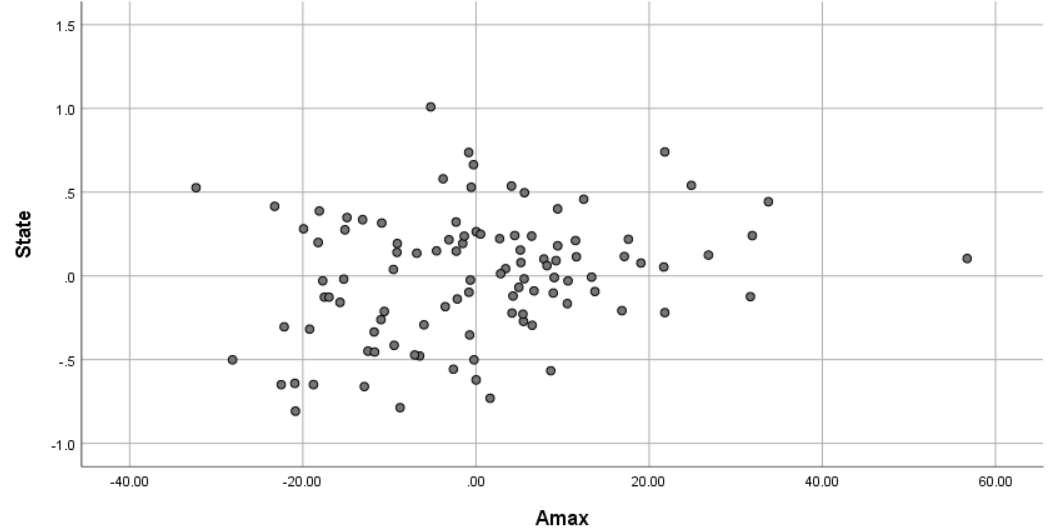


**Supplementary Figure S7.** Partial regression plot of A_max_.


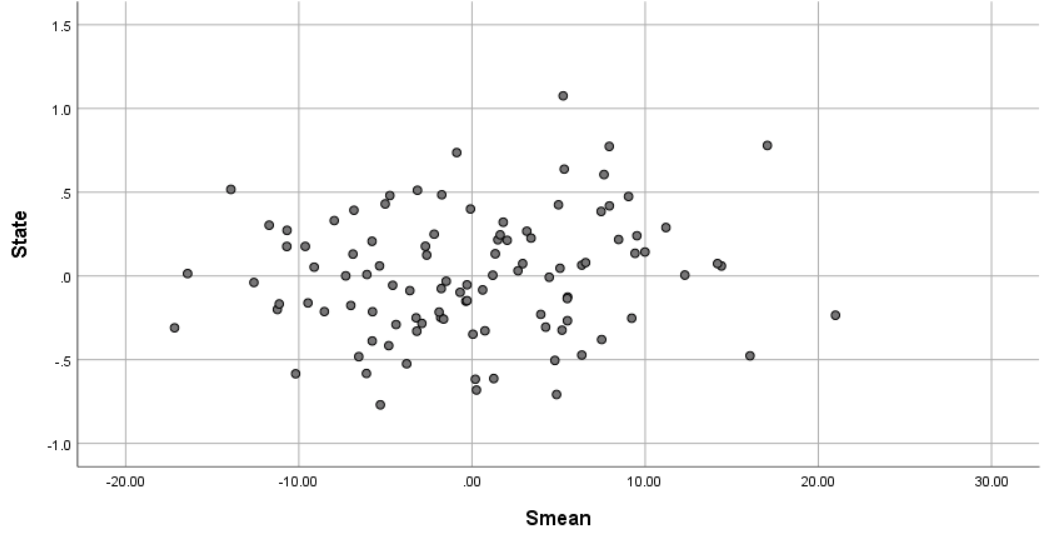


**Supplementary Figure S8.** Partial regression plot of A_mean_.


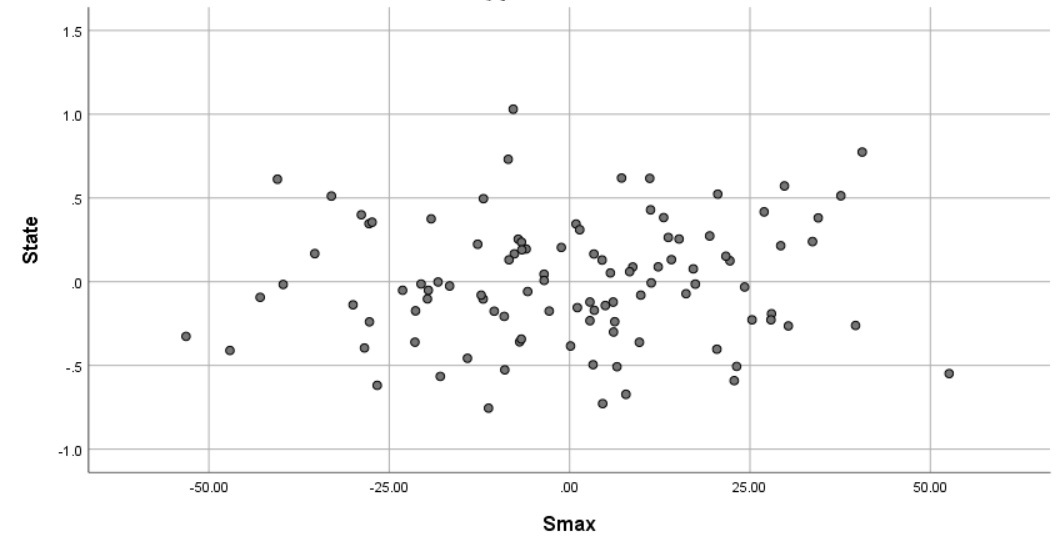


**Supplementary Figure S9.** Partial regression plot of S_max_.

## Supplementary Table

**Supplementary Table S1.** Cumulative importance of features.

| feature | cumulative_importance | feature | cumulative_importance |
| --- | --- | --- | --- |
| Microcalcification | 0.199396 | Age | 0.975831 |
| A_mean_ | 0.384693 | Strain elasticity score | 1 |
| Resistance index | 0.548842 | Orientation | 1 |
| S_mean_ | 0.663646 | Shape | 1 |
| A_max_ | 0.773414 | Margin | 1 |
| S_max_ | 0.840886 | Echo pattern | 1 |
| Size | 0.907351 | Hyperechoic halo | 1 |
| Strain ratio | 0.942598 | Adler classification | 1 |

**Supplementary Table S2.** Performance comparison of XGBOOST and logistic regression.

|  | AUROC | Precision | Recall | F1-score |
| --- | --- | --- | --- | --- |
| **XGBOOST** | | | | |
| CU | 0.53 | 0.83 | 0.42 | 0.56 |
| CU+UE | 0.82 | 0.88 | 0.58 | 0.70 |
| **Logistic regression** | | | | |
| CU | 0.68 | 0.78 | 0.58 | 0.67 |
| CU+UE | 0.72 | 0.80 | 0.67 | 0.73 |

CU: conventional ultrasound UE: ultrasound elastography.
